# Supplementary material for: Oxytocin enhances observational fear in mice
Source: Nat Commun. 2017 Dec 13;8:2102. doi: 10.1038/s41467-017-02279-5 (PMC5727393; doi:10.1038/s41467-017-02279-5)
Supplement: Supplementary file 1 — Supplementary Information [file 41467_2017_2279_MOESM1_ESM.pdf]

SUPPLEMENTARY INFORMATION

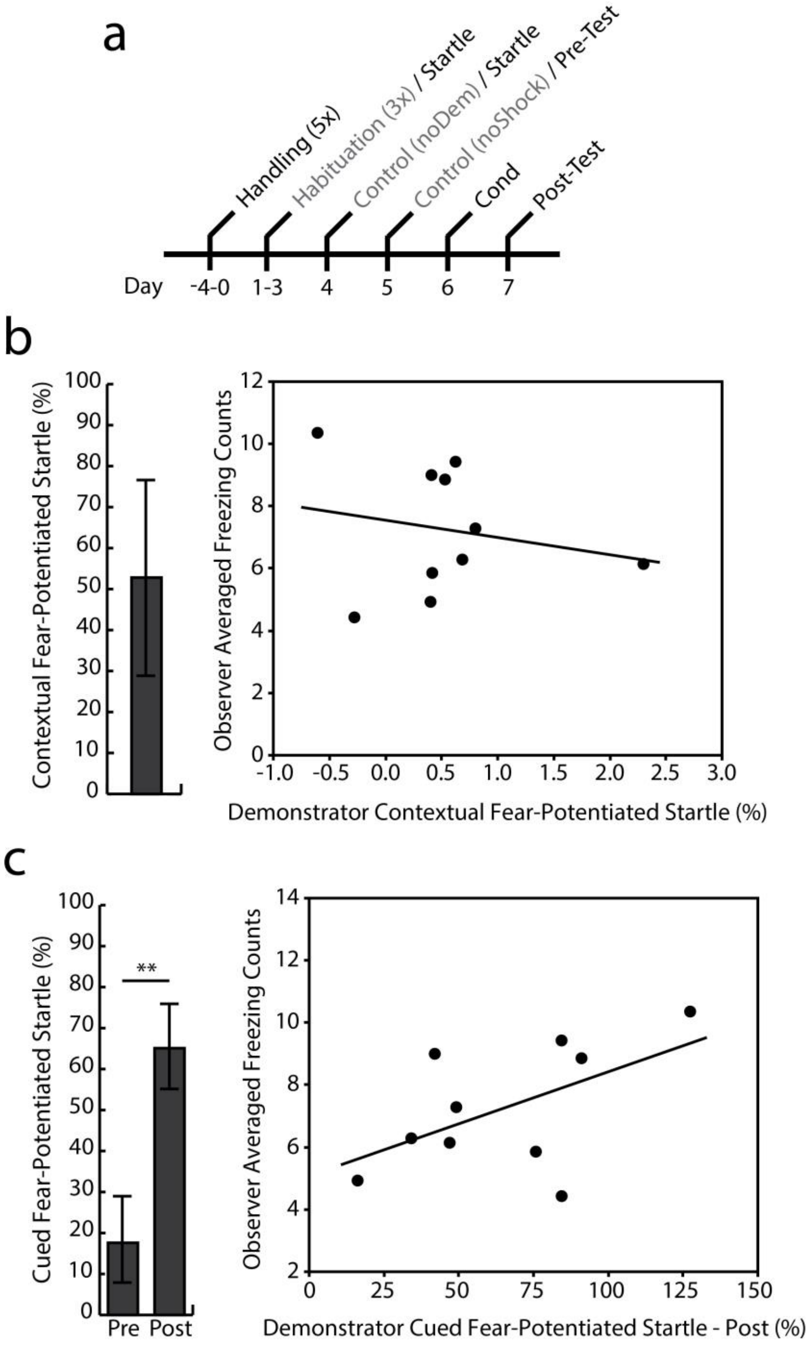

Supplementary Figure 1. Demonstrator fear conditioning. *(**a**) Schematic timeline of demonstrator testing for explicit fear (black), compared with social fear (gray). (**b**) (**left**) Demonstrators (n = 10) express fear-potentiated startle (FPS) to contextual cues, which is not correlated (**right**) with observer freezing during conditioning (Pearson $R^2 = 0.294$, $p = 0.105$). (**c**) (**left**) Demonstrators express significantly elevated cued FPS at post-test (two-tailed student's t test: $t(9) = 3.52$, $p = 0.007$) which shows (**right**) a near-significant correlation with observer freezing during conditioning (Pearson $R^2 = 0.294$, $p = 0.105$). Error bars represent s.e.m. ** $p < 0.01$.*

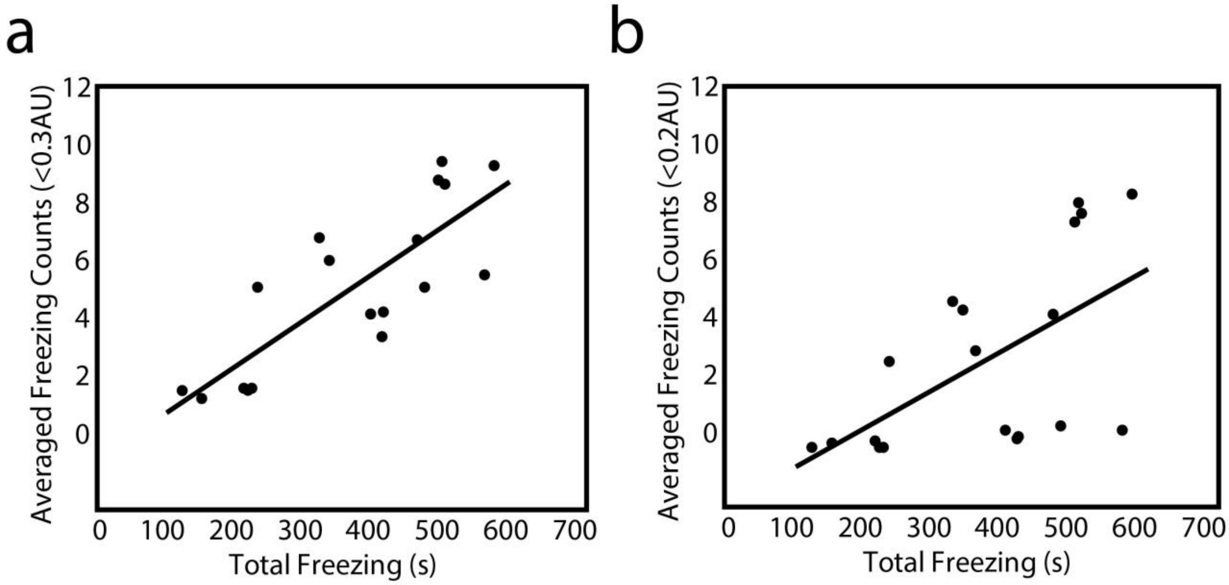

Supplementary Figure 2. Validation of automated freezing behavior. *Automated averaged freezing counts and manual scoring of total freezing behavior are significantly correlated using a (**a**) <0.3AU locomotor activity threshold (Pearson R^2 = 0.65; p <0.0001), compared to a (**b**) <0.2AU threshold (Pearson R^2 = 0.34; p = 0.009). Data represents DREADD animals (n = 19; see Fig 4).*

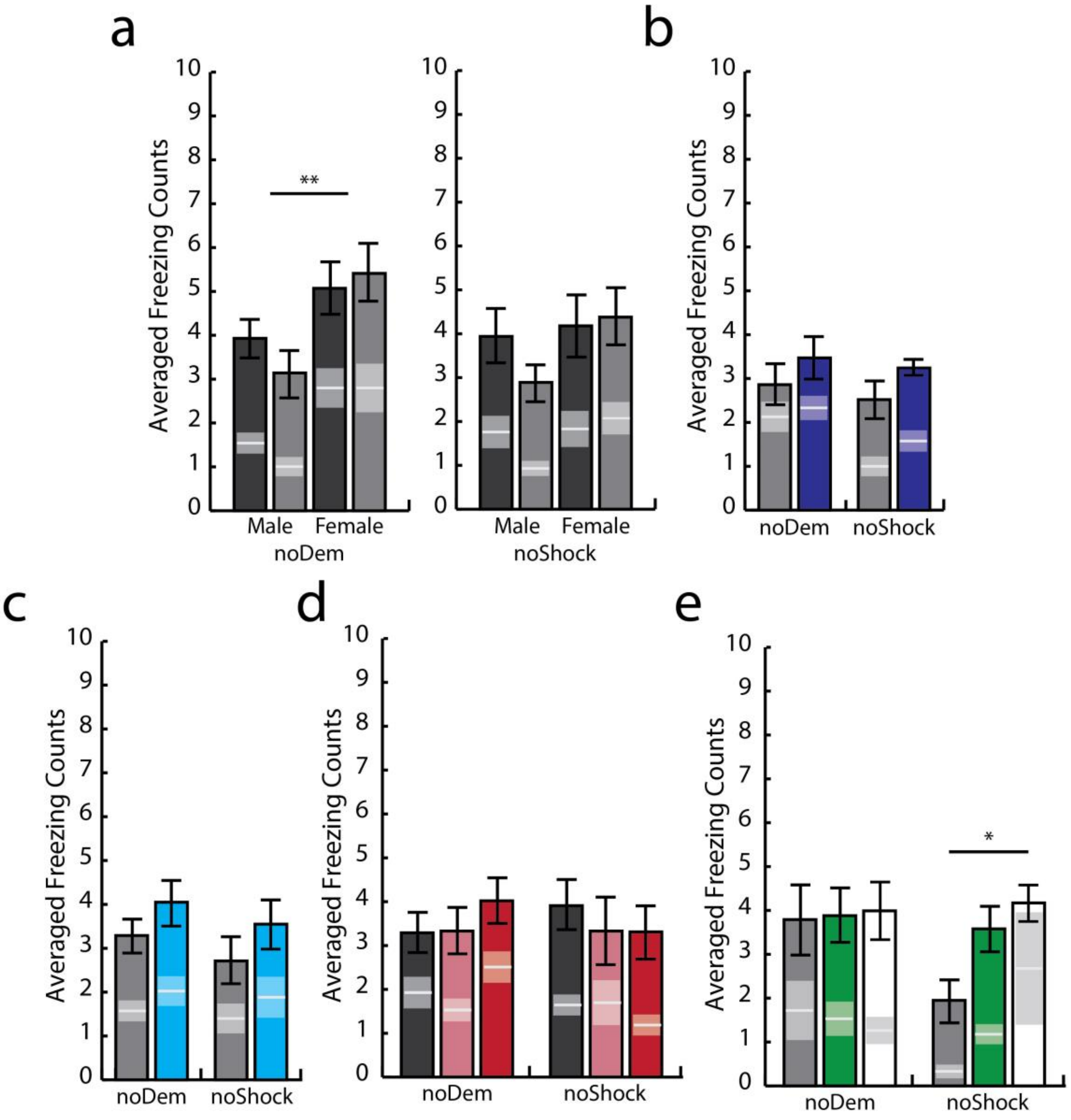

Supplementary Figure 3. Non-specific observer freezing behavior in control conditioning experiments. *(**a**) Non-specific freezing behavior of familiar (black, n = 13) and unfamiliar (gray, n = 13) male and familiar (black, n = 10) and unfamiliar (gray, n = 10) female observer mice during (**left**) noDem (ANOVA: sex effect $F_{1,45}$ = 9.29, p = 0.004; familiarity effect $F_{1,45}$ = 0.166, p = 0.685; sex x familiarity interaction $F_{3,45}$ = 0.990, p = 0.325) and (**right**) noShock (ANOVA: sex effect $F_{1,45}$ = 2.04, p = 0.160; familiarity effect $F_{1,45}$ = 0.49, p = 0.487; interaction effect $F_{3,45}$ = 1.07, p = 0.307) control experiments. (**b**) Non-specific freezing behavior of male observer mice following acute intranasal oxytocin (blue, n = 12) or saline (gray, n = 12) during noDem (two-tailed student's t test: t(23) = 0.88, p = 0.388) and noShock (two-tailed student's t test: t(23) = 1.53, p = 0.152) control experiments. (**c**) Non-specific freezing behavior of male observer mice following chronic intranasal oxytocin (light blue, n = 11) or saline (gray, n = 11) during noDem (two-tailed student's t test: t(21) = 1.16, p = 0.261) and noShock (two-tailed student's t test: t(21) = 1.07, p = 0.296) control experiments. (**d**) Non-specific freezing behavior of male observer mice following saline (I.P., black, n = 12), or 5mg/kg (I.P., pink, n = 12) or 10mg/kg (I.P., red, n = 12) OXTA during noDem (ANOVA: $F_{2,35}$ = 0.65, p = 0.529) and noShock (ANOVA: $F_{2,35}$ = 0.26, p = 0.768) control experiments. (**e**) Non-specific freezing behavior of male (Oxt-Cre/+) observer mice expressing rM3D(Gs) following CNO (I.P., green, n = 10) or saline (I.P., gray, n = 9) or expressing GFP following CNO (I.P., white, n = 10) during noDem (ANOVA: $F_{2,28}$ = 0.02, p = 0.981) and noShock (ANOVA: $F_{2,28}$ = 5.18, p = 0.013) control experiments. Error bars represent s.e.m. * p < 0.05; ** p < 0.01.*

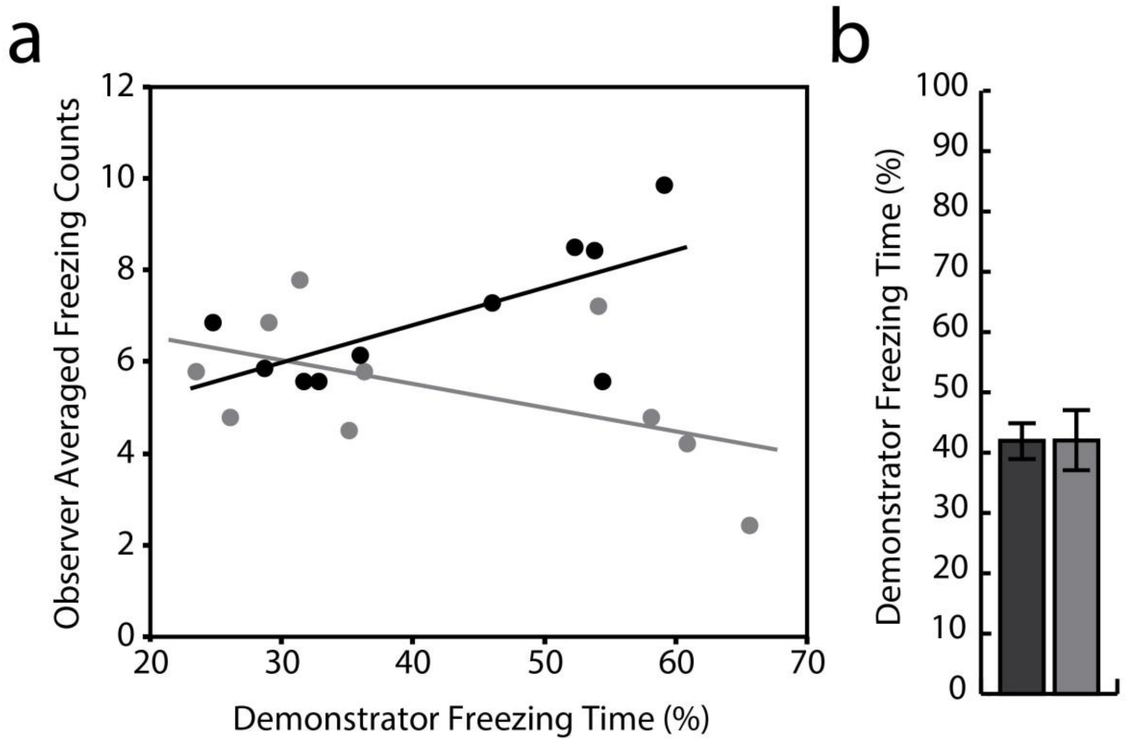

Supplementary Figure 4. Relationship between observer and demonstrator freezing behavior. *(**a**) Observer freezing was correlated with the degree of freezing expressed by the demonstrator in familiar pairs (black, n = 10; Pearson R^2 = 0.455, p = 0.032), but not in unfamiliar pairs (gray, n = 10; Pearson R^2 = 0.264, p = 0.129). (**b**) Familiar and unfamiliar demonstrators exhibited equivalent freezing behavior during conditioning (two-tailed student's t test: t(19) = 0.007, p = 0.997).*

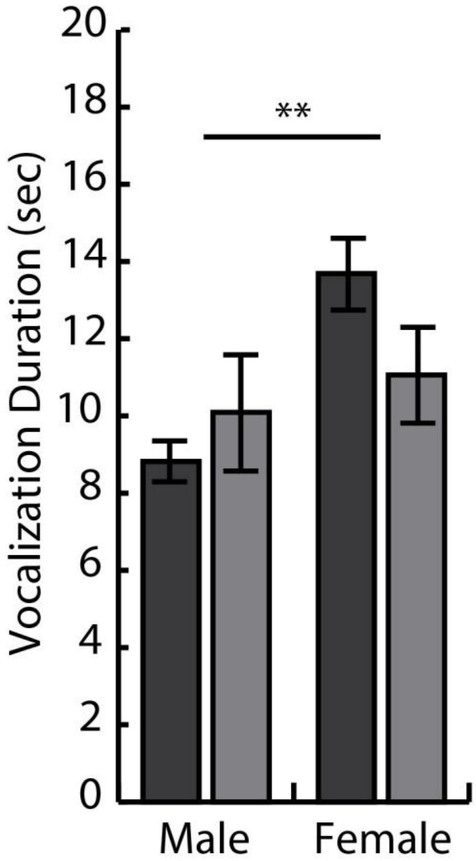

Supplementary Figure 5. Demonstrator vocalizations. *Female demonstrators produced more vocalizations than males (ANOVA: effect of sex* $F_{1,43} = 6.37$, $p = 0.016$*). Error bars represent s.e.m.* ** $p < 0.01$.

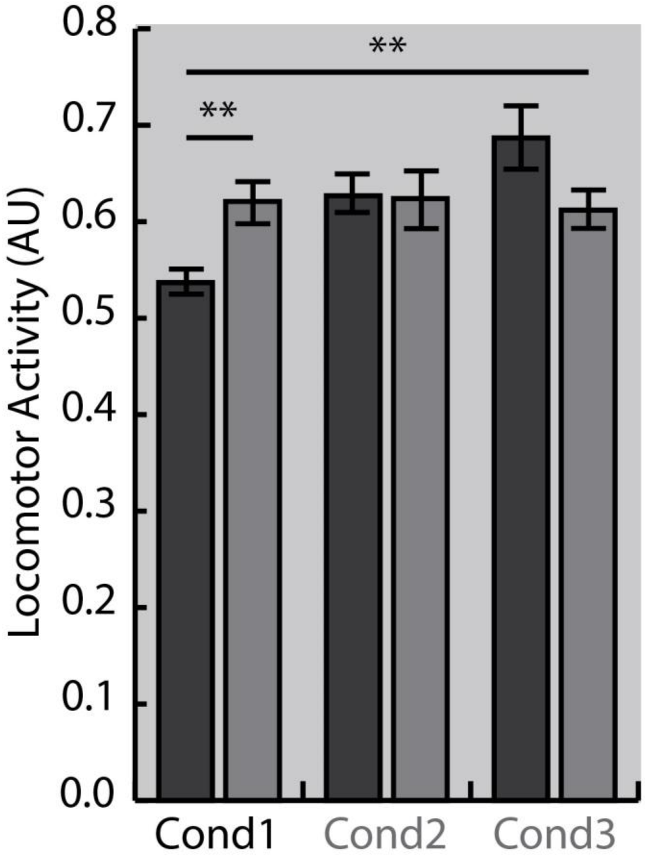

Supplementary Figure 6. Increased locomotor activity across conditioning days in familiar male observers. *Averaged locomotor activity during acclimation of Cond1 was significantly lower in familiar male observers (two-tailed student's t test: $t(25) = 3.08$, $p = 0.008$) compared to unfamiliar observers (see also Fig. 1e) and increased significantly across subsequent conditioning days (repeated measure ANOVA: $F_{5,77} = 3.86$, $p = 0.004$; effect of familiarity $F_{1,77} = 5.96$, $p = 0.017$; effect of day $F_{2,77} = 4.28$, $p = 0.018$; familiarity x day interaction $F_{2,77} = 5.36$, $p = 0.007$). Error bars represent s.e.m. ** $p < 0.01$.*

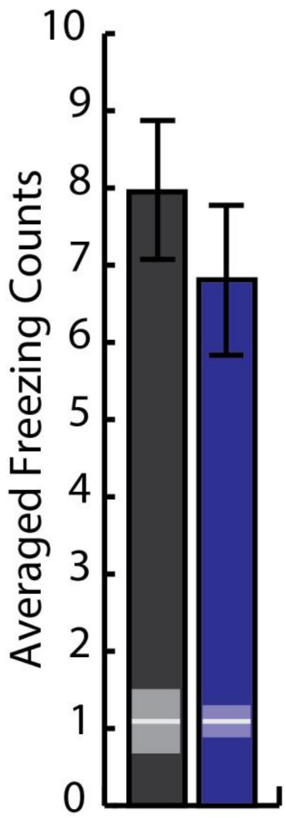

Supplementary Figure 7. Freezing behavior in familiar male mice administered acute intranasal oxytocin. *Compared to saline-treated controls (black, n = 5), familiar male observers treated with acute intranasal oxytocin (20ug/kg; blue, n = 5) thirty minutes prior to demonstrator conditioning showed equivalent freezing (two-tailed student's t test: t(9) = 0.86, p = 0.41). Error bars represent s.e.m.*

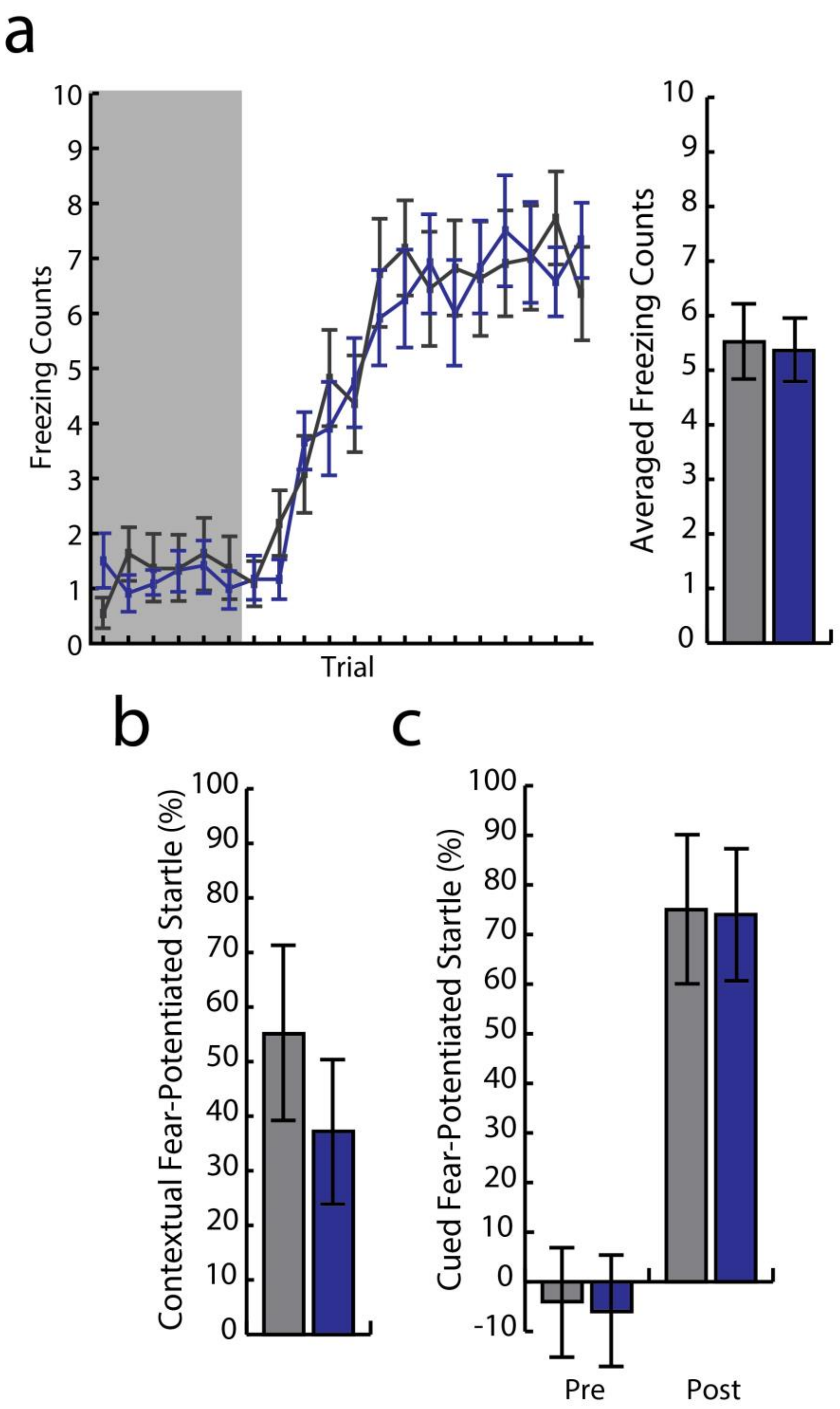

Supplementary Figure 8. Pavlovian fear behaviors of mice following acute intranasal oxytocin. *Compared to saline-treated controls (gray, n = 11), male mice treated with acute intranasal oxytocin (20ug/kg; blue, n = 12) thirty minutes prior to Pavlovian conditioning showed equivalent (**a**) acquisition (**left**, across trials; **right**, averaged) (two-tailed student's t test: t(22) = 0.179, p = 0.859), and (**b**) expression of contextual (two-tailed student's t test: t(22) = 0.859, p = 0.401) and (**c**) cued fear-potentiated startle (post; two-tailed student's t test: t(22) = 0.03, p = 0.975). Error bars represent s.e.m.*

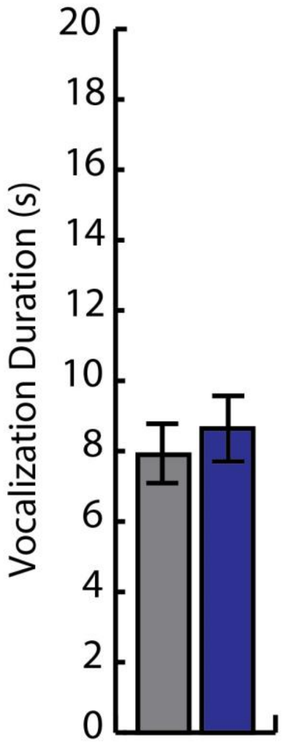

Supplementary Figure 9. Demonstrator vocalizations in acute and chronic intranasal oxytocin experiments. *Demonstrators paired with observer mice administered intranasal oxytocin (acute and chronic; blue; n = 23) produced equivalent vocalization durations, compared to demonstrators paired with saline control mice (gray; n = 23) (ANOVA:* $F_{3,68} = 0.013$*, p = 0.908). Error bars represent s.e.m.*

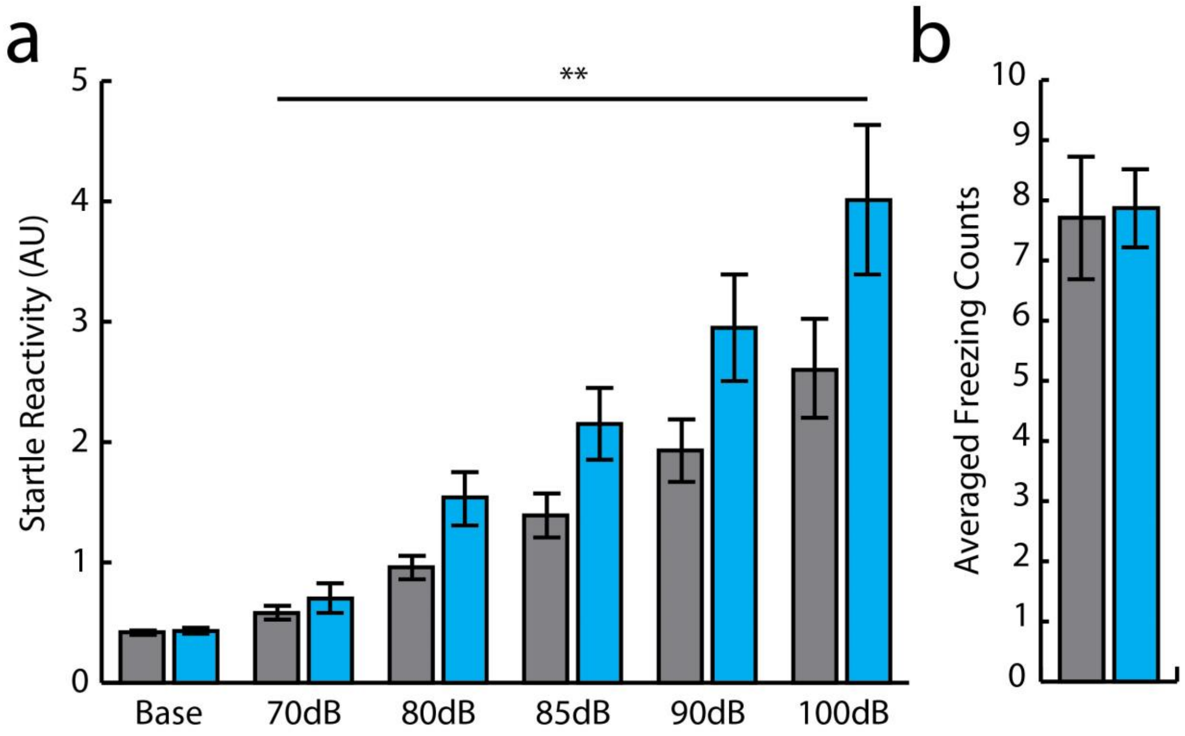

Supplementary Figure 10. Startle reactivity and Pavlovian fear acquisition following chronic intranasal oxytocin. *(**a**) Startle reactivity was significantly elevated in mice administered chronic intranasal OXT (light blue, n = 11) versus saline controls (gray, n = 11) (excluding baseline; ANOVA: $F_{9,109} = 10.578$, $p < 0.0001$; effect of treatment, $F_{1,109} = 8.98$, $p = 0.004$; effect of level, $F_{4,109} = 19.34$, $p < 0.0001$; treatment x level interaction $F_{9,109} = 1.05$, $p = 0.387$). See Fig 3 for testing schedule. (**b**) Averaged freezing during acquisition of Pavlovian fear was equivalent between mice administered chronic intranasal oxytocin (light blue, n = 7) or saline (gray, n = 6) (two-tailed student's t test: $t(12) = 0.134$, $p = 0.896$). Error bars represent s.e.m. ** $p < 0.01$.*

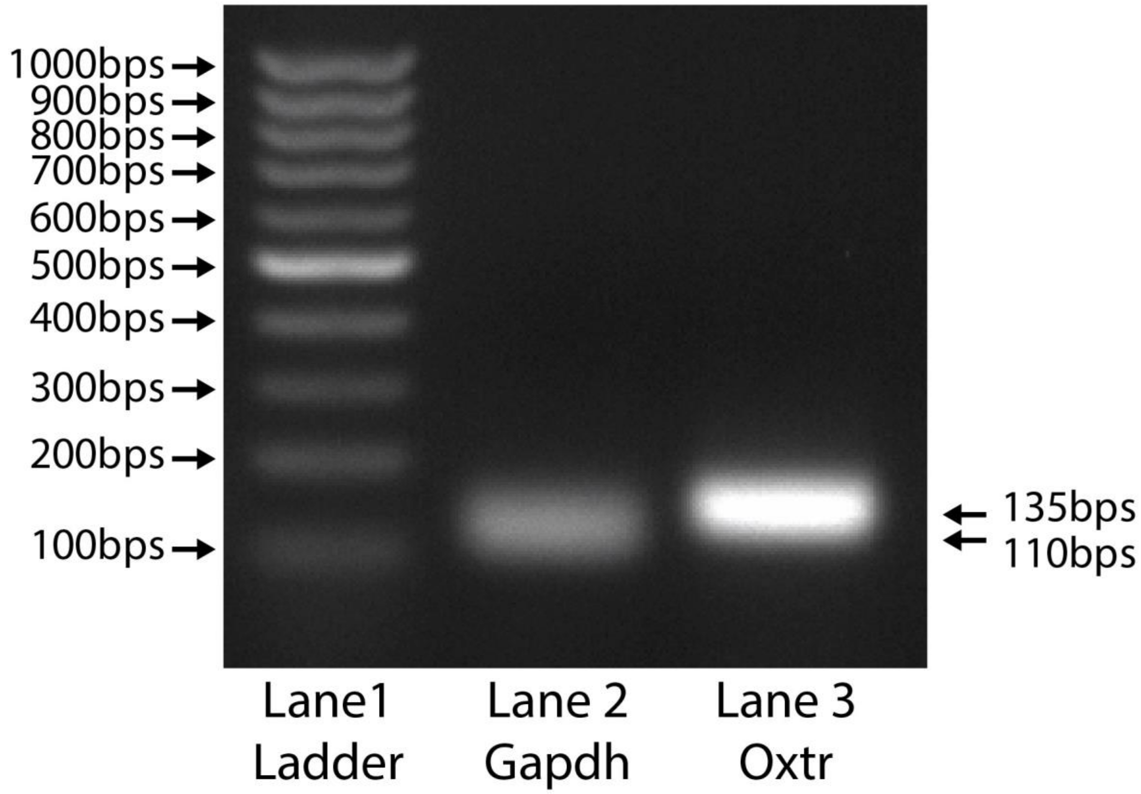

Supplementary Figure 11. Confirmation of qRT-PCR amplicons. *Original gel blot for qRT-PCR amplicons of the Gapdh (110bps, Lane 2) and Oxtr (135bps, Lane 3) transcripts at expected sizes. DNA base pair ladder is shown in Lane 1.*

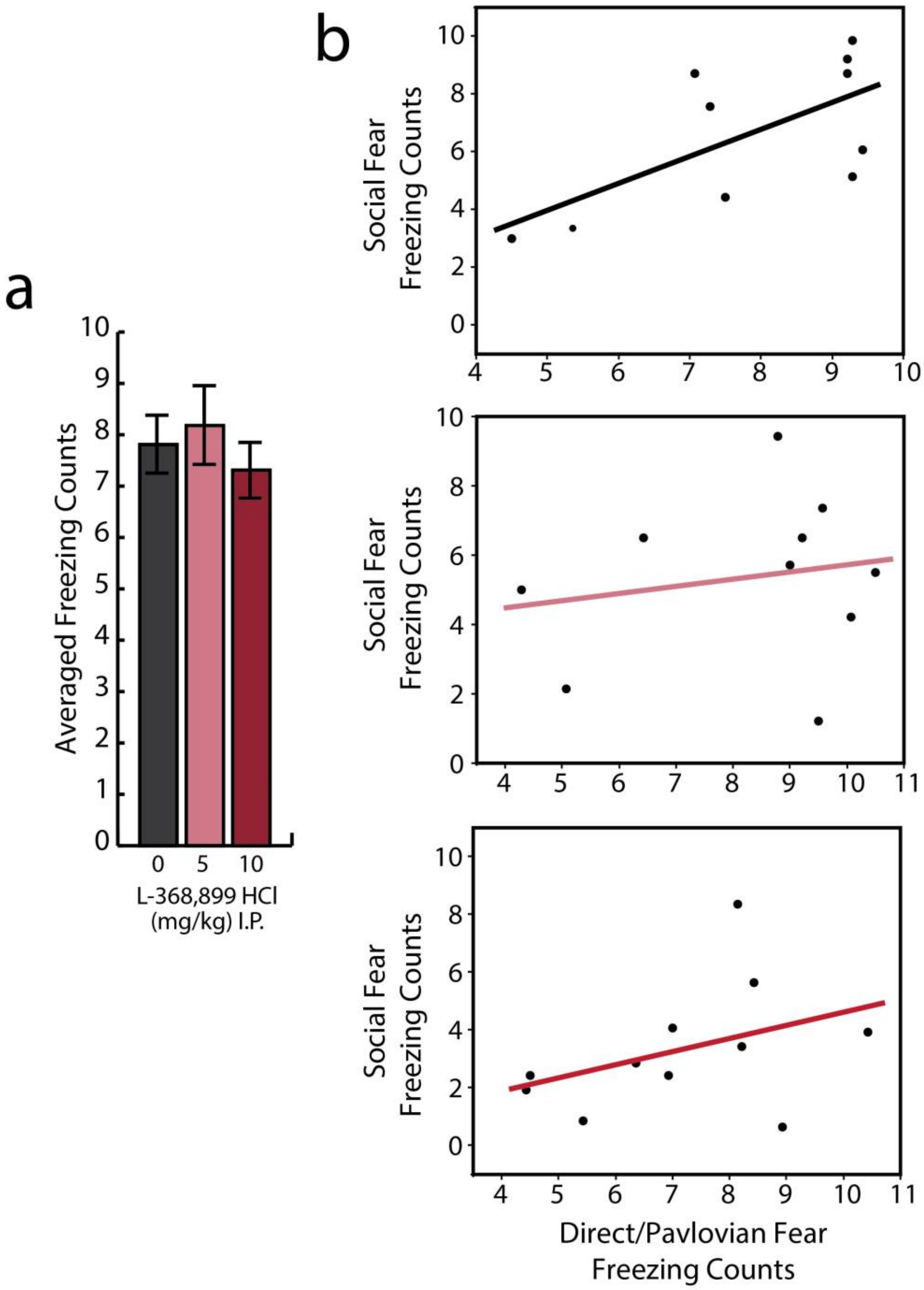

Supplementary Figure 12. Pavlovian fear acquisition and correlations with social fear following systemic oxytocin antagonist. (a) *OXTA (5mg/kg, n = 9; 10mg/kg, n = 12; thirty minutes prior) did not disrupt direct acquisition of Pavlovian fear, compared to saline-treated familiar mice (n = 10) (ANOVA: $F_{2,30} = 0.506$, p = 0.608) (**b**) Saline-treated observer mice displayed a significant correlation between socially and directly acquired fear (upper panel; Pearson $R^2 = 0.434$, p = 0.038), whereas this relationship was absent in mice treated with 5mg/kg (middle panel; Pearson $R^2 = 0.035$, p = 0.606) or 10mg/kg (bottom panel; Pearson $R^2 = 0.160$, p = 0.194) OXTA. Error bars represent s.e.m.*

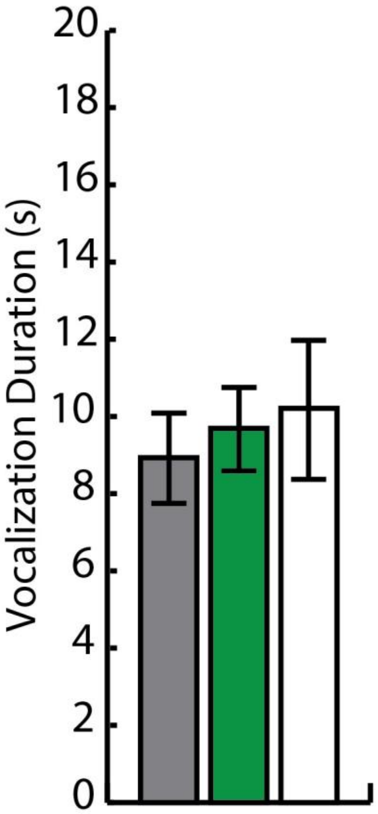

***Supplementary Figure 13. Demonstrator vocalizations in chemogenetic experiments**.
Demonstrators elicited equivalent vocalization durations for observer mice expressing rM3D(Gs) administered saline (gray; n = 9) or CNO (3mg/kg, I.P.; green; n = 10), and observer mice not expressing rM3D(Gs) administered CNO (white; n = 10) (ANOVA: $F_{2,29}$ = 0.215, p = 0.807). Error bars represent s.e.m.*

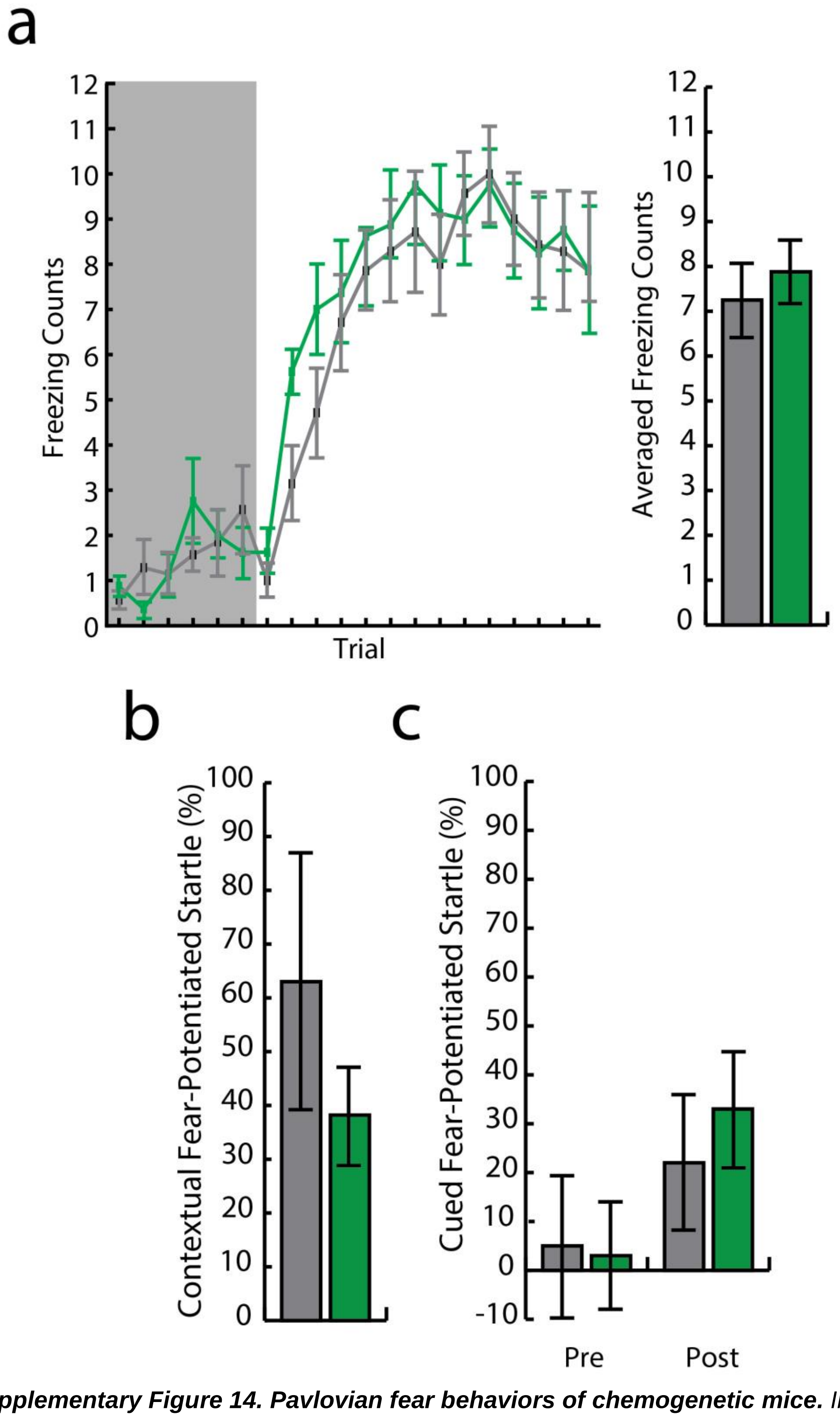

Supplementary Figure 14. Pavlovian fear behaviors of chemogenetic mice. *In observer mice expressing the rM3D(Gs) receptor, saline (gray, n = 7) and CNO (3mg/kg, I.P.; green, n = 8) treatment thirty minutes prior to conditioning produced similar (**a**) acquisition (**left**, across trials; **right**, averaged) (two-tailed student's t test: t(14) = 1.380, p = 0.190), and expression of (**b**) contextual (two-tailed student's t test: t(14) = 0.969, p = 0.362) and (**c**) cue-specific fear (post; two-tailed student's t test: t(14) = 0.429, p = 0.675). Error bars represent s.e.m.*

Supplementary Table 1. Litter sizes for experimental groups.

	Fam/ Male	Unfam/ Male	Fam/ Female	Unfam/ Female	Unfam/ Acute OXT	Unfam/ Acute SAL	Unfam/ Chronic OXT	Unfam/ Chronic SAL	Unfam/ CNO	Unfam/ SAL	Fam/ SAL	Fam/ 5mg/kg OXTA	Fam/ 10mg/kg OXTA
**Average**	**8.00**	**8.15**	**8.30**	**7.60**	*	*	**7.63**	**5.66**	**7.80**	**7.70**	**7.50**	**7.66**	**7.90**
**S.E.**	0.73	0.37	0.33	0.58	*	*	0.43	0.92	0.53	0.58	0.43	0.16	0.58
**Sig?**	n.s.		n.s.		n.s.		n.s.		n.s.		n.s.		

* Data not available.

Supplementary Movie 1. Freezing behavior of a familiar observer male mouse (bottom) in response to the conditioning of a demonstrator conspecific (top).

Supplementary Movie 2. Escape behavior of a familiar observer male mouse in response to the conditioning of a demonstrator conspecific (during Cond(open))
