# Supplementary material for: Oxytocin enhances observational fear in mice
Source: Nat Commun. 2017 Dec 13;8:2102. doi: 10.1038/s41467-017-02279-5 (PMC5727393; doi:10.1038/s41467-017-02279-5)
Supplement: Supplementary file 3 — Description of Additional Supplementary Files [file 41467_2017_2279_MOESM3_ESM.pdf]

Description of Additional Supplementary Files

File Name: Supplementary Movie 1
Description: Freezing behavior of a familiar observer male mouse (bottom) in response to the conditioning of a demonstrator conspecific (top).

File Name: Supplementary Movie 2
Description: Escape behavior of a familiar observer male mouse in response to the conditioning of a demonstrator conspecific (during Cond(open))
